# Precarbonization Facilitated Closed Pores Formation and Surface Graphitization on Bamboo-Derived Hard Carbon to Improve Sodium Storage Performance

**DOI:** 10.3390/ma19081538

**Published:** 2026-04-12

**Authors:** Gao-Yang Bai, Wen-Jing Sun, Zu-Wei Yin, Ze-Bin Pan, Chuan-Wei Wang, Yao Zhou, Jun-Tao Li

**Affiliations:** College of Energy, Xiamen University, Xiamen 361005, China

**Keywords:** sodium-ion batteries, hard carbon anode, precarbonization, closed pore, surface structure

## Abstract

Hard carbon (HC) was considered as a promising anode candidate for Na-ion batteries, due to its ability of efficient Na-ion storage. Bamboo-derived HC has the advantages of sustainability, environmental benefits and low cost, which are crucial for advancing the commercialization of SIBs technology. Precarbonization has been reported as a method to improve the electrochemical performance of HC anodes derived from various precursors, while the underlying mechanism behind why precarbonization improved the electrochemical performance of bamboo-derived HC has not been studied in detail. Herein, the effect of precarbonization on electrochemical behavior, bulk and surface structure, and surface composition was comprehensively explored. The results revealed that the improved reversible capacity was attributed to the increased closed pores for extra Na-ion storage, increased surface N content and decreased oxygen content for Na-ion absorption/desorption; the improved cycling stability was ascribed to the reduced surface oxygen and C-O content leading to suppressed side reactions, while the improved surface graphitization degree contributed to rate capability enhancement. This work clarified the role of precarbonization in improving the hard carbon anode for Na-ion batteries, which will be helpful to the commercialization of hard carbon materials.

## 1. Introduction

The development demand of large-scale energy storage devices and low-speed transportation applications make sodium-ion batteries (SIBs) a promising technology due to their low cost and abundant natural sodium resources [1,2]. The dominated anode material for Li-ion batteries (LIBs), graphite, exhibits unsatisfactory kinetics in SIBs, due to the larger size of Na^+^ (0.102 nm) comparing with Li^+^ (0.076 nm) and the limited carbon interlayer spacing [3,4]. Hard carbon (HC) recently emerged as a promising anode candidate material for SIBs, due to its advantages [5]: (1) a disordered microstructure with expanded interlayer spacing (0.37–0.42 nm) enabling efficient Na^+^ storage; (2) defect-rich architectures providing enough absorption/desorption sites for substantial pseudocapacitive storage; and (3) lower volume expansion rate (<10%) compared with alloy-based anodes (e.g., >300% volume expansion in Sb anodes).

Among HC anodes derived from different precursors, biomass-derived hard carbon exhibits the advantages of sustainability, environmental benefits and low cost, which are crucial for advancing the commercialization of SIBs technology [6,7]. However, the application of biomass-derived HC still faces several issues [8]: (1) The bulk structure of HC, especially pore structure, plays a critical role in its electrochemical performance, yet controlling its pore structure has remained a challenge. (2) The complex composition and irregular structure of biomass materials often lead to inconsistent performance in the carbonization products. Therefore, simultaneously regulating the bulk and surface structure of HC to enhance its sodium storage performance remains a key challenge in current research.

Many methods have been developed to regulate the bulk and surface structure of HC [5,8], such as pre-oxidation, CO_2_ etching, particle size control, surface functional group regulation, element doping, and surface coating etc. However, current research predominantly focuses on fusible precursors (i.e., sucrose, asphalt, etc.) to regulate their melting behaviors, or utilizes rigid biomass to generate open pores for adsorption [9,10,11]. These strategies inevitably induce abundant open defects that severely compromise the initial Coulombic efficiency (ICE). For highly aligned rigid biomass like bamboo, achieving superior performance through a purely thermal-driven solid-state rearrangement to upgrade open defects into closed pores remains a critical scientific gap.

To bridge this gap, in this study, we explored the structural and kinetic evolution of bamboo-derived hard carbon through a tailored, purely thermal precarbonization strategy (i.e., 800 °C for deep devolatilization followed by 1400 °C for pore closure). The results indicate that precarbonization drives the topological transition of open defects into more closed pores to significantly boost the plateau capacity. It also leads to enhanced surface graphitization, lower oxygen content and higher nitrogen content on the surface, which results in higher reversible capacity at both the slope voltage region of >0.1 V and plateau voltage region of <0.1 V, lower charge transfer resistance and interfacial resistance, and further improved cycling stability and rate performance.

## 2. Experimental

### 2.1. Material Preparation

The bamboo-derived hard carbon materials were provided by Fujian Keda New Energy Technology Co., Ltd. (Sanming, China). The baseline sample underwent acid washing followed by carbonization at 1400 °C under N_2_ atmosphere. In comparison, the precarbonization sample has an additional 800 °C precarbonization process before the carbonization at 1400 °C.

### 2.2. Material Characterization

The morphology and microstructure of the hard carbon materials were observed using scanning electron microscopy (SEM, ZEISS Sigma, Carl Zeiss, Oberkochen, Germany) and high-resolution transmission electron microscopy (HRTEM, Tecnai F30 TWIN, Thermo Fisher Scientific, Waltham, MA, USA). The particle size distribution was measured by a laser particle size analyzer (BT-9300ST, Bettersize Instruments, Dandong, China). Crystal structure was analyzed by X-ray diffraction (XRD, Rigaku Ultima IV, Rigaku, Tokyo, Japan) with Cu Kα radiation. The graphitization degree was evaluated using Raman spectroscopy (WITec Alpha 300R, WITec, Ulm, Germany, 532 nm laser). The nanostructure, including open and closed pores, was probed by small-angle X-ray scattering (SAXS, Xenocs Xeuss 3.0 HR, Xenocs, Grenoble, France). The structural parameters of the closed pores were quantitatively fitted based on standard scattering invariant models. Specific surface area and pore size distribution were determined by N_2_ and CO_2_ physisorption at 77 K and 273 K, respectively, using a Micromeritics TriStar II 3020 analyzer (Micromeritics Instrument Corporation, Norcross, GA, USA). The cross-sectional areas of N_2_ and CO_2_ molecules were taken as 0.162 nm^2^ and 0.170 nm^2^. The specific surface area was calculated via the Brunauer–Emmett–Teller (BET) method and the precise pore size distributions were derived utilizing non-local density functional theory (NLDFT) models. Surface heteroatom content and bonding configurations were characterized by X-ray photoelectron spectroscopy (XPS, Thermo Fisher Escalab Xi+, Thermo Fisher Scientific, Waltham, MA, USA). Diffuse reflectance infrared Fourier transform spectroscopy (DRIFTS) was performed on a Fourier transform infrared spectrometer (Bruker Vertex 70V, Bruker, Ettlingen, Germany).

### 2.3. Electrochemical Tests

The electrochemical performance of the as-prepared hard carbon samples was evaluated in CR2025-type coin cells using sodium metal as the counter electrode. A homogeneous slurry was prepared by mixing the active material, carbon black, and polyvinylidene fluoride (PVDF) binder in a mass ratio of 80:10:10. The slurry was uniformly coated onto a copper foil current collector and dried under vacuum at 100 °C for 12 h. The dried electrode was cut into disks with a diameter of 12 mm, yielding an active material mass loading of approximately 2–3 mg cm^−2^. The electrolyte was 1.0 M sodium hexafluorophosphate (NaPF_6_) in a mixture of ethylene carbonate and dimethyl carbonate (EC/DMC, 1:1 by volume). A Celgard 2400 membrane was used as the separator. Galvanostatic charge/discharge tests were conducted within a voltage window of 0–2.0 V (vs. Na^+^/Na). Cyclic voltammetry (CV) measurements were carried out on a CHI660D electrochemical workstation (Shanghai Chenhua Instrument Co., Ltd., Shanghai, China) at a scan rate of 0.05 mV s^−1^. Electrochemical impedance spectroscopy (EIS) was conducted using Princeton VERSASTATV3 electrochemical workstation (Princeton Applied Research, Oak Ridge, TN, USA). Long-term cycling performance and rate capability tests were performed using a LAND 2001A battery test system (Wuhan LAND Electronics, Wuhan, China), and 1 C = 350 mAh g^−1^.

## 3. Results and Discussions

### 3.1. Electrochemical Performance

**Figure 1:**
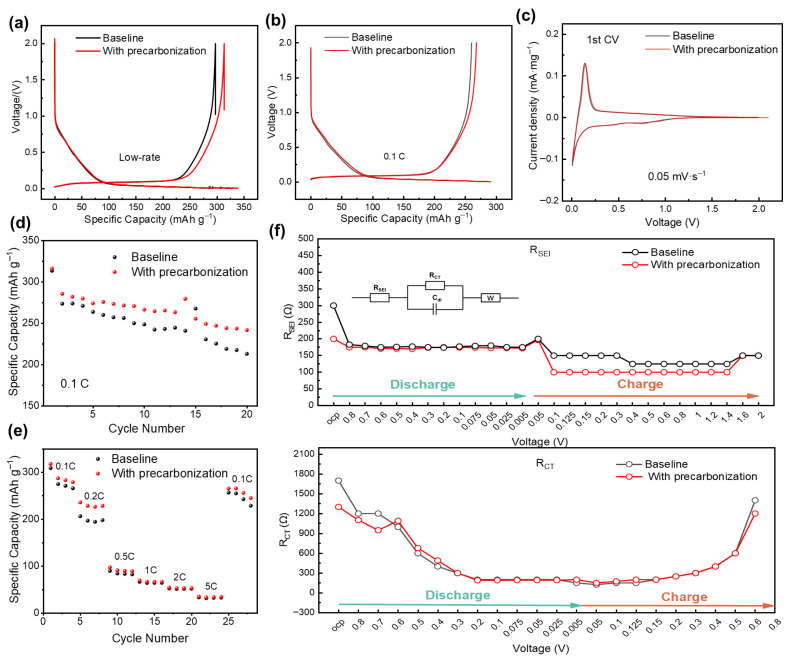
Initial charge–discharge curves of HC anodes at (**a**) operation condition with low-rate discharge and 0.1 C charge, and (**b**) charge and discharge at 0.1 C. (**c**) Cyclic voltammetry (CV) curves of HC anodes with a scan rate of 0.05 mV/s. (**d**) Cycling stability of HC anodes at 0.1 C. (**e**) Rate capability of HC anodes. (**f**) Solid electrolyte interphase resistance (up) and charge transfer resistance (bottom) evolution with different voltages derived from in situ EIS results during first charge–discharge at 0.1 C.

To study the effect of precarbonization on the electrochemical charge–discharge behavior of hard carbon anodes, galvanostatic charge–discharge and cyclic voltammetry (CV) tests were conducted. Figure 1a shows the initial charge–discharge curves of HC anodes without precarbonization (denoted as baseline) and with precarbonization at an operation condition with low-rate discharge (0.1 C with subsequent 0.08 C, 0.05 C, 0.03 C, 0.01 C and 0.005 C at voltage region of <0.1 V and 0.1 C charge rate. At voltage region of <0.1 V, the Na^+^ storage is predominantly associated with pores filling and insertion into graphitized layer [12]. Since the slow kinetics of pore filling and Na^+^ insertion into graphitized layer, such a low-rate discharge can make the capacity of hard carbons exhibit as high as possible. As listed in Table 1, it can be seen that the HC anode with precarbonization leads to both a higher discharge (338.54 vs. 326.95 mAh g^−1^) and charge capacity (313.31 vs. 297.1 mAh g^−1^), as well as a higher initial Coulombic efficiency (92.55% vs. 90.87%). At a operate condition of 0.1 C charge rate and 0.1 C discharge rate (Figure 1b), precarbonization leads to similar higher discharge (291.20 vs. 286.06 mAh g^−1^) and charge capacity (267.84 vs. 260.9 mAh g^−1^), as well as a higher initial Coulombic efficiency (91.98% vs. 90.92%) (Table 1). The difference between the first discharge capacity at low-rate and 0.1 C is 47.34 and 40.89 mAh g^−1^ for HC anode with precarbonization and baseline, respectively.

The 1st, 2nd and 3rd charge–discharge curves of HC anodes without and with precarbonization at a low-rate discharge condition are displayed in Figure S1a and Figure S1c, respectively. Combining the corresponding dQ/dV curves in Figure S1b,d, the first-three-cycle comparison of charge–discharge curves (Figure 1c and Figure S2) and CV curves (Figures S3 and S4) for baseline and HC anode with precarbonization, it can be concluded that the precarbonization makes no obvious difference on the Na-ion storage kinetic mechanism. The discharge capacities at the voltage of >0.1 V and <0.1 V for HC anodes were compared (Table 2) to explore the role of precarbonization in improving the capacity reversibility. At an operation condition with low-rate discharge, both the baseline and precarbonization samples exhibit capacity decay at the voltage region of >0.1 V and <0.1 V, while both the capacity at the voltage region of >0.1 V and <0.1 V keep steady during the 2nd and 3rd cycle. For the baseline sample without precarbonization, the reversible capacity is ~66 mAh g^−1^ and 232 mAh g^−1^ for the voltage region of >0.1 V and <0.1 V, respectively. As a comparison, for the precarbonization sample, the reversible capacity is ~74 mAh g^−1^ and 240 mAh g^−1^ for the voltage region of >0.1 V and <0.1 V, respectively. At both the slope region of >0.1 V and plateau region of <0.1 V, the HC anode with precarbonization displays a ~8 mAh g^−1^ higher reversible capacity than the baseline sample. At 0.1 C, a similar trend was observed; precarbonization induces higher reversible capacity for the hard carbon anode, while precarbonization only leads to a ~5 mAh g^−1^ higher capacity than the baseline sample, which is lower than that of ~8 mAh g^−1^ under the low-rate discharge condition. It can be contributed to the sluggish kinetic of hard carbon anode.

Figure 1d,e exhibit the cycling stability and rate capability of HC anodes, respectively. It can be obviously observed that precarbonization has a positive effect on improving both cycling stability and rate capability. The in situ electrochemical impedance spectroscopy (EIS) results during first charge–discharge and second discharge were shown in Figure S5. The evolution of resistance of solid electrolyte interphase (R_SEI_) and charge transfer (R_ct_) during first cycle was shown in Figure 1f (up) and (bottom) respectively, which were fitted based on the in situ EIS results in Figure S5 and equivalent circuit diagram shown in the inset of Figure 1f (up). During the first charge–discharge process, the precarbonization sample demonstrates an overall slightly lower R_SEI_ and R_ct_ than the baseline sample, indicating the precarbonization leads to improved kinetic of hard carbon anode, which is consistent with the cycling stability and rate capability demonstrated in Figure 1d,e.

From above, precarbonization sample has a similar Na-ion storage kinetic process with the baseline sample without precarbonization, while the precarbonization sample exhibits a higher reversible capacity at both the voltage region of >0.1 V and <0.1 V, improved cycling stability and rate capability, which is related with the lower resistance of solid electrolyte interphase (R_SEI_) and charge transfer (R_ct_) during charge–discharge.

### 3.2. Structure Analysis

**Figure 2:**
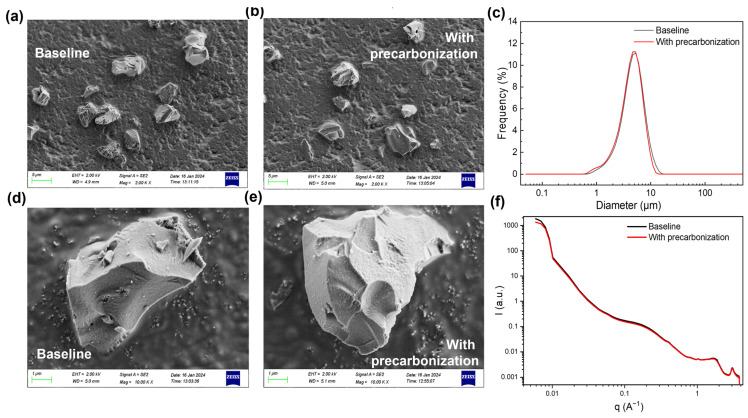
SEM images of HC anodes (**a**,**d**) without precarbonization and (**b**,**e**) with precarbonization. (**c**) Particle size distribution analysis of HC anodes. (**f**) SAXS spectrum of HC anodes.

To investigate the underlying mechanism that precarbonization improve the electrochemical performance of hard carbon anode, both bulk and surface structure analysis was conducted. As displayed in Figure 2a,b,d,e, SEM images indicate that precarbonization has no distinct effect on the particle size of hard carbon anode. Such results coincided with the particle size distribution analysis using the laser particle analyzer (Figure 2c), while the D_50_ of baseline is 5.075 μm and D_50_ of precarbonization sample is a similar 4.84 μm.

**Figure 3:**
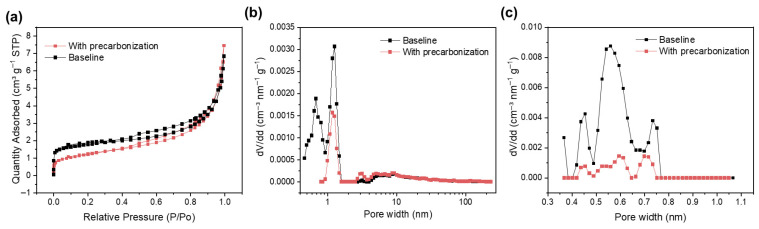
(**a**) Nitrogen adsorption/desorption isotherms and (**b**) corresponding pore size distributions of HC anodes with and without precarbonization. Pore size distributions of HC anodes (**c**) with precarbonization and without precarbonization based on CO_2_ adsorption/desorption isotherms.

The pore structure was studied using small-angle X-ray scattering (SAXS) and Brunauer–Emmett–Teller (BET) specific surface area analysis. The SAXS was tested based on the X-ray scattering on the pore structure, which is a powerful tool for the micropore (<2 nm) and mesopore (2–50 nm), including both the open pores and closed pores. The BET test based on N_2_ adsorption/desorption mainly provides the pore structure analysis with width >1 nm, while the BET test based on CO_2_ adsorption/desorption supplementarily provides the pore structure analysis with width <1 nm. The N_2_ isothermal adsorption and desorption curves of HC anode without and with precarbonization was shown in Figure 3a. Both the isotherms show type I and type II structures with H_4_ hysteresis, which is consistent with the expected adsorption behavior of micro-mesoporous materials [11]. The BET equation was used to calculate the total specific surface area of SCs, with detailed information presented in Table 3. The surface area of hard carbon was reduced from 6.4464 m^2^ g^−1^ to 4.3303 m^2^ g^−1^ with a precarbonization process. The volume contributed by pores with width of 1–2 nm was decreased after precarbonization, but the quantity of pores with width of 2–50 nm was slightly increased (Figure 3b), which is consistent with the increased median pore diameter of 10.6539 nm for precarbonizatin sample, while the baseline shows a median pore diameter of 6.5666 nm. For the CO_2_ isothermal adsorption/desorption curves for pores with width <1 nm, the HC anodes with or without precarbonization shows similar pore diameter distribution (Figure 3c), but the whole pore volume of precarbonization sample is smaller than the baseline sample (Figure S7), which coincides with the lower surface area of precarbonization sample (6.065 m^2^ g^−1^) than baseline (18.421 m^2^ g^−1^).

Both the BET tests based on N_2_ and CO_2_ adsorption/desorption indicate that precarbonization results in the decrease in open pores. Combining with the similar SAXS results of HC anodes with and without precarbonization manifesting similar overall pores volume, it can be concluded that precarbonization leads to the increase in closed pores in the HC anode. To rigorously quantify this structural evolution, the SAXS profiles were fitted based on standard scattering invariant models. As evidenced by the quantitative structural parameters detailed in the Supporting Information (Figure S6 and Table S1), the average closed pore radius increases from 9.04 Å to 10.3 Å after precarbonization. Since closed pores contributed to Na^+^ storage under 0.1 V [12,13], the increased closed pores and enlarged pore dimensions in HC anode with precarbonization will lead to increased reversible capacity at the voltage region of <0.1 V, which is in accord with the electrochemical results (Figure 1a,b and Table 2).

**Figure 4:**
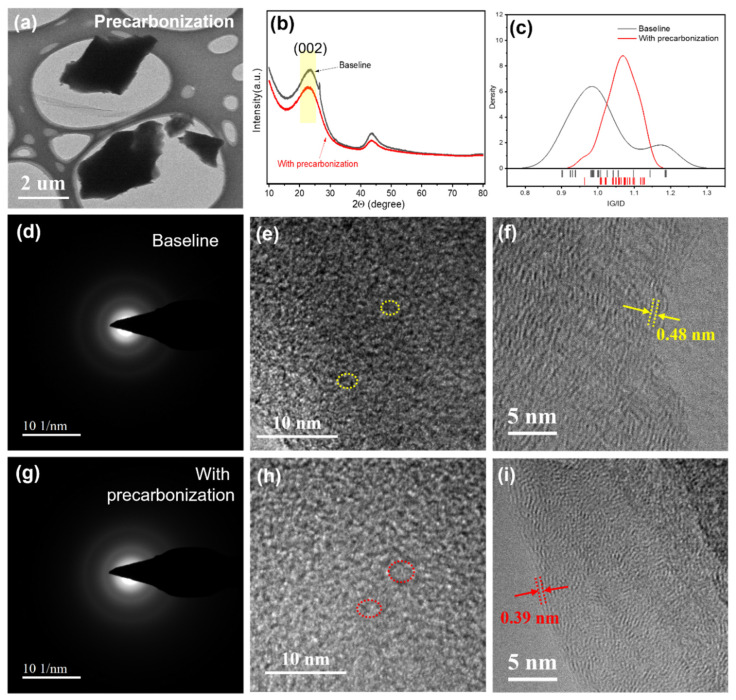
(**a**) XRD patterns and (**b**) surface graphitization comparison of HC anodes with/without precarbonization. TEM images of HC anodes (**c**,**g**–**i**) with precarbonization and (**d**–**f**) without precarbonization. The dotted yellow and red circles in (**e**) and (**h**), respectively, denote the presence of closed micropores.

To investigate the crystalline structure of HC anodes, transmission electron microscope (TEM), X-ray diffraction (XRD) and Raman spectrum were employed. TEM images demonstrate a similar micron-level size of HC anodes with and without precarbonization (Figure 4a and Figure S8a). Since no obvious electron diffraction (ED) points were observed in both baseline (Figure 4d) and HC anode after precarbonization (Figure 4g), low crystallinity degree of hard carbon anodes can be confirmed, which manifests the (002) peak in the XRD originated from the average crystalline layer including the closed micropores (Figure 4b). The XRD results indicate a (002) peak shift to small angle of the HC anode after precarbonization (Figure 4b), manifesting an increase in average crystalline layer space. Specifically, the calculated d_002_ value (based on Bragg’s law, λ = 0.15406 nm) increased from 0.377 nm for the baseline sample to 0.391 nm for the precarbonized sample. The above SAXS and BET results indicate increased closed pores after precarbonization, which coincides with the XRD and ED results (Figure 4e,h). The surface crystalline structure was revealed by Raman spectra and TEM images (Figure 4c,f,i). The Raman spectra at 30 different points for HC anodes without and with precarbonization are shown in Figure S9. For carbon materials, there are obvious peaks corresponding to D-band and G-band, whose intensity represents the disorder degree and graphitization degree (crystalline degree) respectively. Based on the Raman spectra in Figure S9, a statistic of ratio of G-band peak intensity to D-band peak intensity is displayed in Figure 4c. An average higher graphitization degree of precarbonization sample can be distinctly observed. Typical TEM images of surface structure of HC anodes without and with precarbonization are exhibited in Figure 4f and Figure 4i, respectively. In the same scale bar of 5 nm, it can be observed that the crystalline space in precarbonization sample is lower than that in baseline. Specifically, a representative surface lattice spacing of 0.48 nm can be observed in the baseline sample (Figure 4f), while a narrower lattice spacing of 0.39 nm was shown in the precarbonization sample (Figure 4i).

### 3.3. Surface Composition Analysis

**Figure 5:**
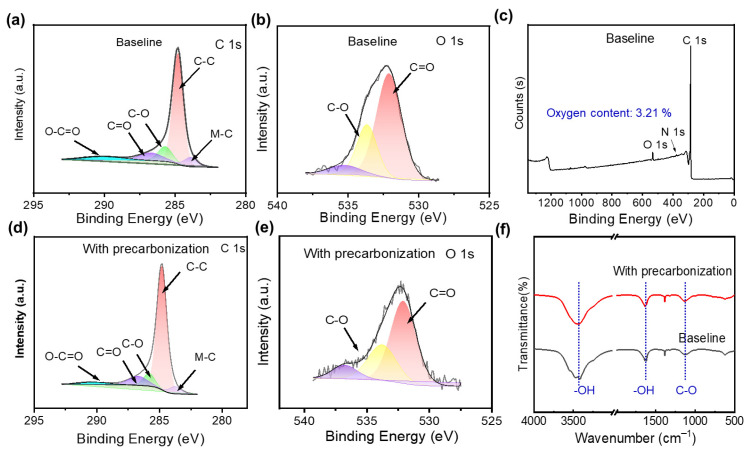
XPS of (**a**,**d**) C1s and (**b**,**e**) O1s of HC anodes (**a**,**b**) without precarbonization and (**d**,**e**) with precarbonization. (**c**) XPS full spectrum of HC anodes without precarbonization. (**f**) FTIR spectra of HC anodes with/without precarbonization.

X-ray photoelectron spectroscopy (XPS), nitrogen–oxygen analyzer and Fourier Transform Infrared Spectroscopy (FTIR) were conducted to explore the effect of precarbonization on the surface composition. From the XPS C1s results of baseline (Figure 5a) and precarbonization sample (Figure 5d), it can be observed that the ratio of C-C band was increased after precarbonization, which is consistent with the increased graphitization results evidenced by Raman and TEM results (Figure 4c,f,i). Simultaneously, C-O peak intensity shown in both C 1s (Figure 5a,d) and O 1s (Figure 5b,e) was decreased after precarbonization. The surface oxygen content analysis derived from XPS full spectra (Figure 5c and Figure S10) indicate an oxygen content decrease after precarbonization (2.74% for precarbonization vs. 3.21% for baseline), which is consistent with the XPS C1s and O1s analysis. The surface oxygen content decrease was further confirmed by FTIR spectra, in which -OH and C-O group was reduced after precarbonization (Figure 5f). However, the oxygen content analysis of the bulk structure in hard carbon anodes indicate that no obvious difference induced by the precarbonization process (Table 4), while both the nitrogen content on the surface and in the bulk were improved (bulk: 0.133% for precarbonization sample vs. 0.105% for baseline; surface: 0.37% precarbonization sample vs. 0.24% for baseline).

### 3.4. Electrochemical Performance Improvement Mechanism

The above results manifest that the precarbonization leads to electrochemical performance improvement as below: (1) higher reversible capacity at both the slope voltage region of >0.1 V and plateau region of <0.1 V; (2) improved cycling stability and rate capability; and (3) lower resistance of solid electrolyte interphase (R_SEI_) and charge transfer (R_ct_) during charge–discharge. The structure and composition change in hard carbon anode induced by precarbonization: (1) more closed pores; (2) reduced surface area; (3) increased surface graphitization degree; (4) decreased surface C-O and oxygen content; and (5) increased N content both on the surface and in the bulk.

Combining the electrochemical behavior and structure/composition analysis, the electrochemical performance improvement mechanism by precarbonization can be concluded: (1) more closed pores provided extra Na-ion storage space [12,13,14], leading to higher reversible capacity at the plateau voltage region of <0.1 V; (2) the higher reversible capacity at the slope voltage of >0.1 V can be attributed to the increased surface N content and decreased oxygen content, since the capacity at the slope voltage region of >0.1 V always attributed to Na-ion absorption/desorption [15,16]; (3) since surface C-O can induce side reactions with ester electrolyte (i.e., EC) [17], reduced surface C-O content and surface area will suppress the interfacial side reactions, further leading to improved cycling stability; and (4) the higher graphitization degree of the surface carbon layer will lead to higher electronic conductivity [18,19], further leading to improved rate capability.

## 4. Conclusions

In summary, precarbonization was successfully employed to improve the reversible capacity, cycling stability and rate capability of bamboo-derived hard carbon anodes for Na-ion batteries. Combining characterizations of bulk structure, surface structure and surface composition, the improved reversible capacity was attributed to the increased closed pores, increased surface N content and decreased oxygen content; the improved cycling stability was ascribed to the reduced surface oxygen and C-O content; the improved surface graphitization degree contributed to rate capability enhancement. This work provided a detailed mechanism study behind why precarbonization improves the electrochemical performance of hard carbon anodes for Na-ion batteries, which will also give clues for other carbon materials design.

## Figures and Tables

**Table 1:** First-cycle charge/discharge capacities and Coulombic efficiencies of HC anodes with/without precarbonization at different operate conditions.

Sample	Operate Condition	1st Discharge Capacity (mAh g^−1^)	1st Charge Capacity (mAh g^−1^)	Initial Coulombic Efficiency (%)
Baseline	Low-rate discharge	326.95	297.1	90.87
	0.1 C	286.06	260.9	90.92
With precarbonization	Low-rate discharge	338.54	313.31	92.55
	0.1 C	291.20	267.84	91.98

**Table 2:** Comparison of 1st, 2nd and 3rd discharge capacities at the voltage of >0.1 V and <0.1 V of HC anodes with/without precarbonization at different operate conditions.

Sample	Operate Condition	1st Discharge Capacity (mAh g^−1^)		2nd Discharge Capacity (mAh g^−1^)		3rd Discharge Capacity (mAh g^−1^)	
		>0.1 V	<0.1 V	>0.1 V	<0.1 V	>0.1 V	<0.1 V
Baseline	Low-rate discharge	84.93	242.02	66.63	232.39	65.53	231.02
	0.1 C	83.13	202.93	65.57	194.88	65.34	190.28
With precarbonization	Low-rate discharge	88.19	250.35	74.05	240.98	73.05	240.32
	0.1 C	88.84	202.36	74.08	200.88	73.09	195.59

**Table 3:** Surface area and median pore width of HC anodes based on N_2_ and CO_2_ adsorption/desorption.

Sample	Surface Area from N_2_ BET (m^2^ g^−1^)	Average Pore Width (4V/A, N_2_, nm)	Surface Area from CO_2_ BET (m^2^ g^−1^)	Median Pore Width (H-K, CO_2_, nm)
Baseline	6.45	6.57	18.42	0.73
With precarbonization	4.33	10.65	6.07	0.77

**Table 4:** The bulk and surface O and N element content of HC anodes with/without precarbonization.

Sample	Bulk Content		Surface Content	
	O Content (wt.%)	N Content (wt.%)	O Content (wt.%)	N Content (wt.%)
Baseline	0.561	0.105	3.21	0.24
With precarbonization	0.561	0.133	2.74	0.37

## Data Availability

The original contributions presented in this study are included in the article/Supplementary Materials. Further inquiries can be directed to the corresponding authors.

## Supplementary Materials

The following supporting information can be downloaded at: https://www.mdpi.com/article/10.3390/ma19081538/s1.
