# Supplementary material for: Precarbonization Facilitated Closed Pores Formation and Surface Graphitization on Bamboo-Derived Hard Carbon to Improve Sodium Storage Performance
Source: Materials (Basel). 2026 Apr 12;19(8):1538. doi: 10.3390/ma19081538 (PMC13117378; doi:10.3390/ma19081538)
Supplement: Supplementary file 1 [file materials-19-01538-s001.zip › materials-4232398-supplementary.pdf]

Support Information

for

Pre-carbonization Facilitated Closed Pores Formation and Surface Graphilization on Bamboo-derived Hard Carbon to Improve Sodium Storage Performance

Gao-Yang Bai[1], Wen-Jing Sun[1], Zu-Wei Yin[1,*], Ze-Bin Pan[1], Chuan-Wei Wang[1], Yao Zhou[1], Jun-Tao Li[1,*]

[1] College of Energy, Xiamen University, Xiamen 361005, China

Email: yinzuwei@xmu.edu.cn (Z. W. Yin), jtli@xmu.edu.cn (J. T. Li)

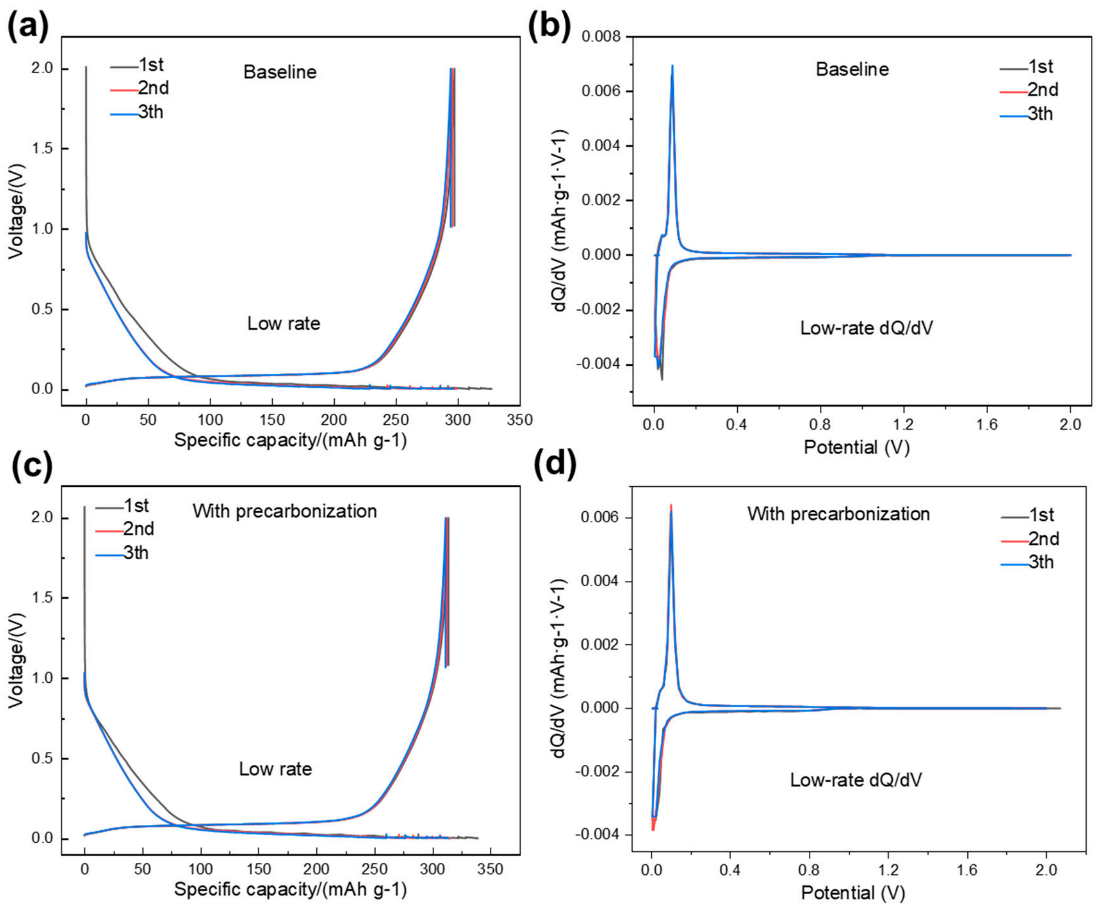

Figure S1. The 1st, 2nd and 3rd charge-discharge curves of HC anodes (a) without and (c) with precarbonization at an operate condition with low-rate discharge and 0.1 C charge rate, and the corresponding dQ/dV curves: (b) without and (d) with precarbonization.

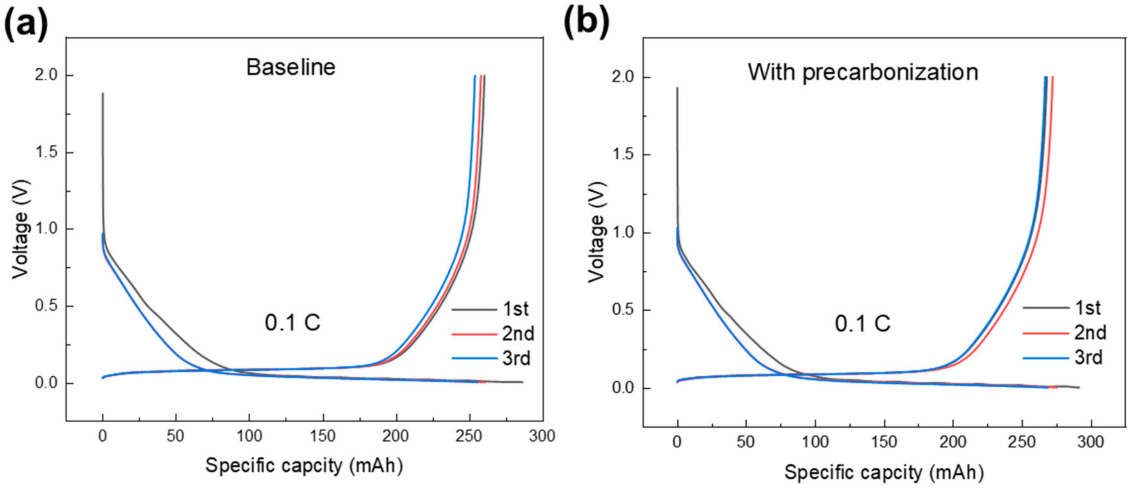

Figure S2. The 1st, 2nd and 3rd charge-discharge curves of HC anodes (a) without and (b) with precarbonization at 0.1 C.

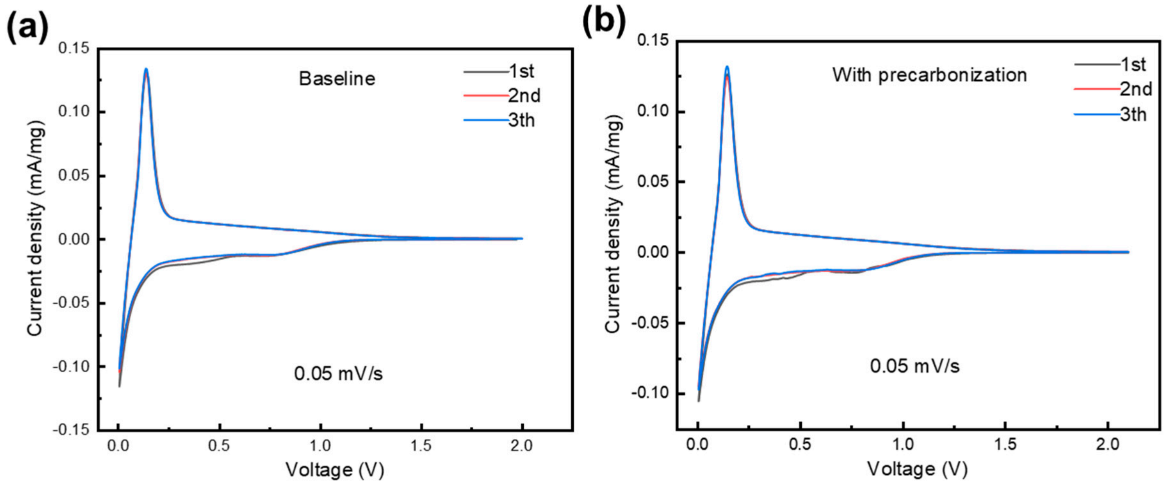

Figure S3. The 1st, 2nd and 3rd CV curves of HC anodes (a) without and (b) with precarbonization at a scan rate of 0.05 mV/s.

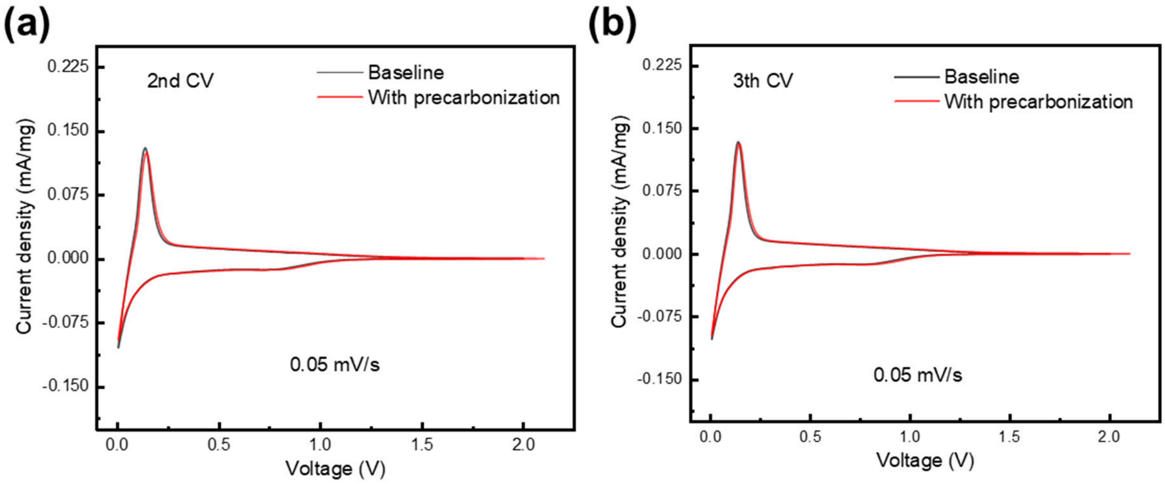

Figure S4. Comparison of (a) 2nd and (b) 3rd CV curves of HC anodes (a) without and (b) with precarbonization.

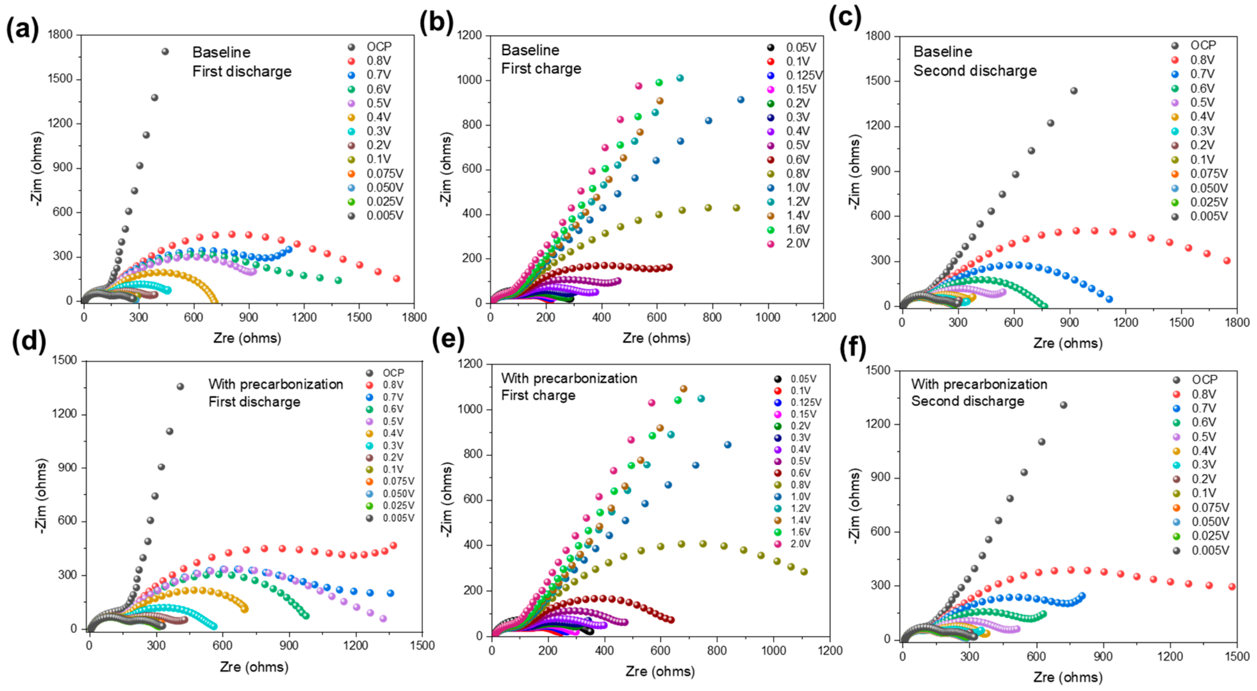

Figure S5. In-situ EIS results of HC anodes (a-c) without and (d-f) with precarbonization: (a, d) First discharge, (b, e) first charge, (c, f) second discharge.

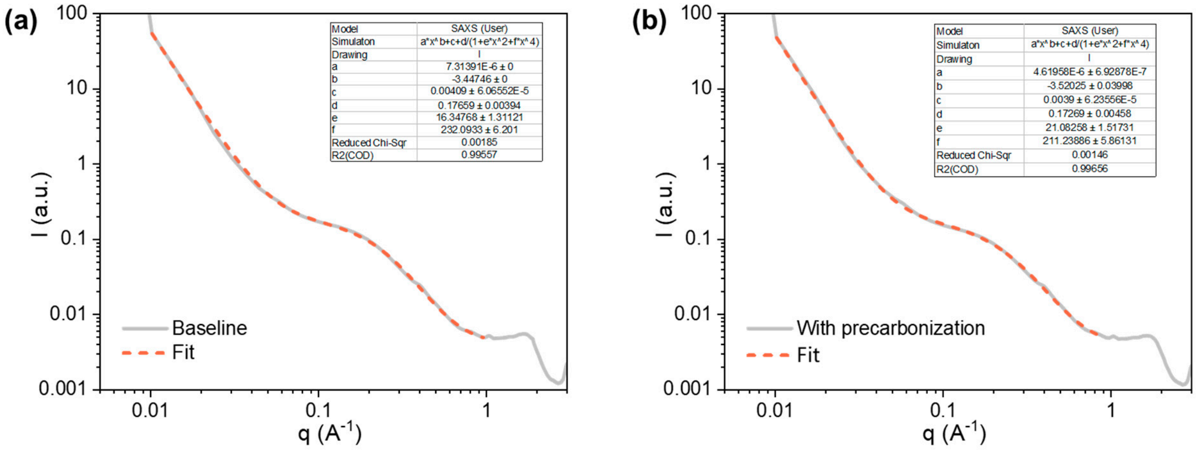

Figure S6. SAXS scattering profiles and the corresponding theoretical fitting curves of (a) baseline and (b) pre-carbonized bamboo-derived hard carbon samples.

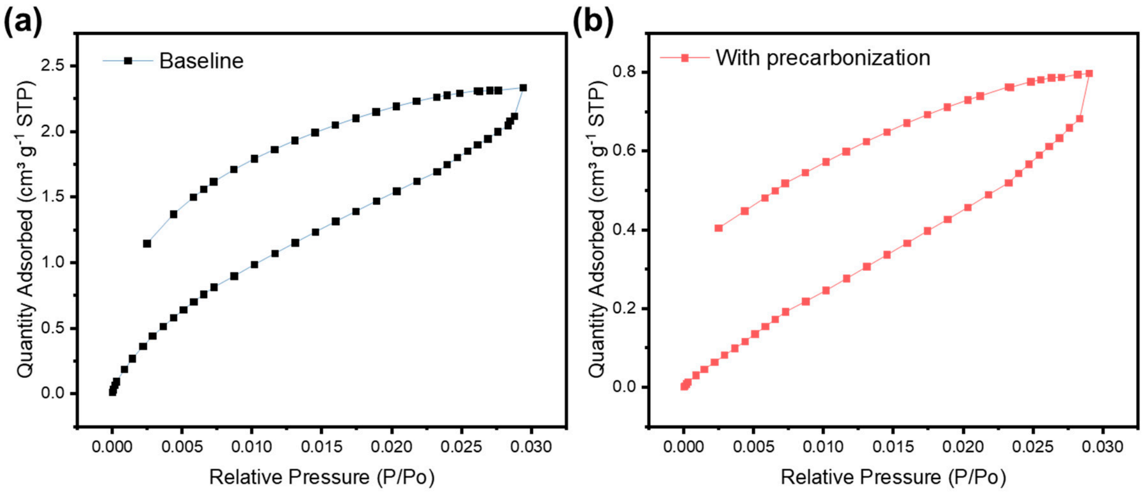

Figure S7. CO_2 adsorption/desorption isotherms of HC anodes (a) without precarbonization and (b) with precarbonization.

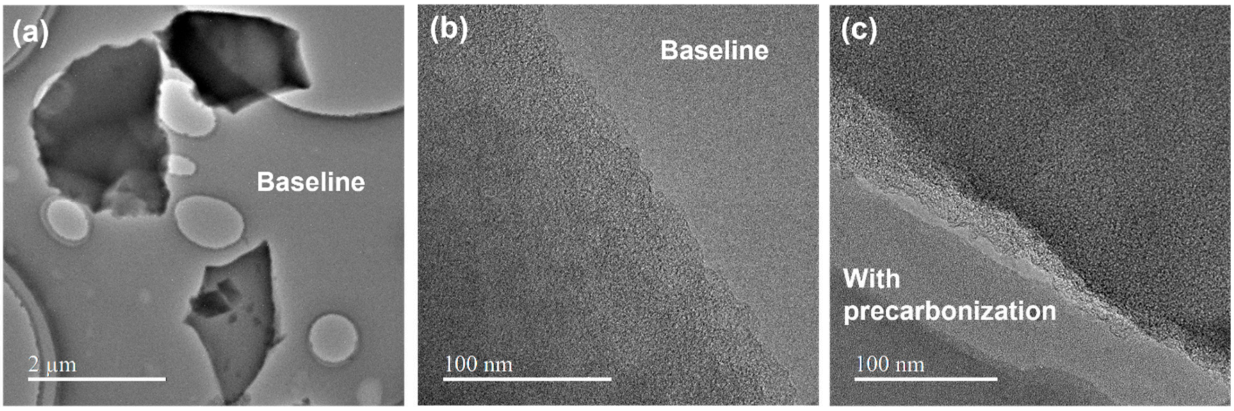

Figure S8. TEM images of HC anodes (a, c) with and (b) without precarbonization

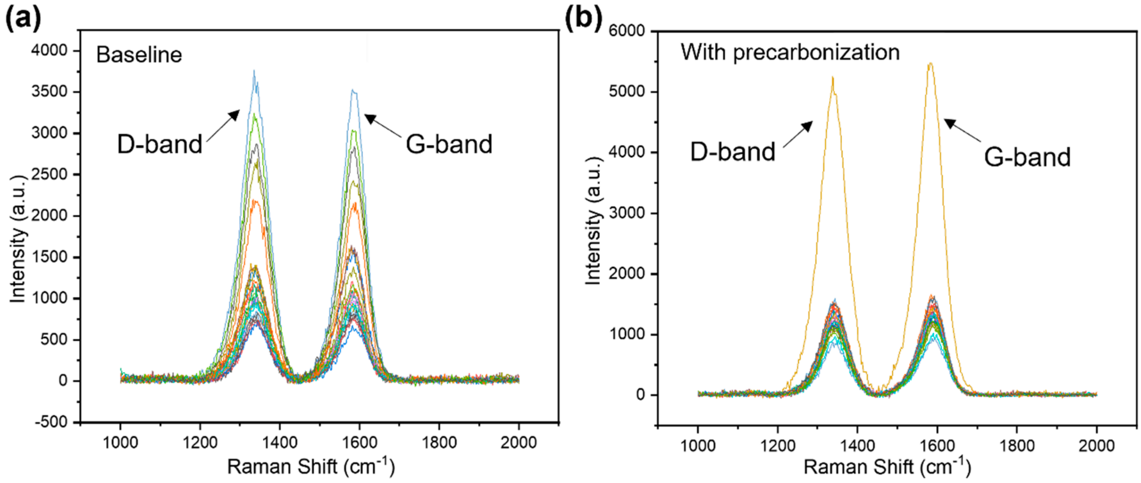

Figure S9. Raman spectra at 30 different points for of HC anodes (a) without and (b) with precarbonization.

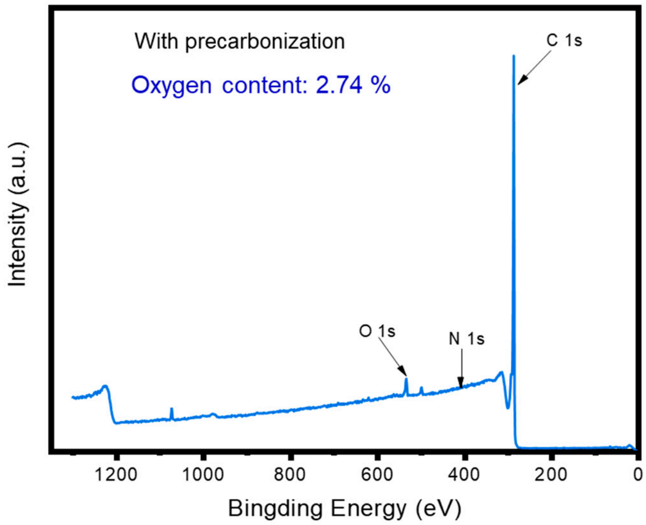

Figure S10. XPS full spectrum of HC anode with precarbonization.

Table S1. Quantitative structural parameters of the closed pores in bamboo-derived hard carbon samples derived from SAXS fitting results.

Sample	Average Pore Radius (Å)	Inter-pore Distance (Å)
Baseline	9.04	50.9
With precarbonization	10.3	53.5

Table S2. Performance comparison with recently reported hard carbon anodes.

Precursor	N2 BET Surface Area ($m^2 g^{-1}$)	Reversible Capacity (mAh g^{-1})	Current Density (mA g^{-1})	ICE (%)	Method	Reference
**Bamboo**	**4.33**	**291.2**	**35**	**92.6**	**Precarbonization**	**This work**
Bamboo	19.21	281.6	30	70.4	Acid-leaching + Precarbonization	[6]
Asphalt	2.20	333.7	30	77.2	Precarbonization	[9]
Coffee Grounds	40.03	339.5	30	80.2	Mg-assisted + Precarbonization	[10]
Sucrose	20.60	374.0	20	85.0	Precarbonization	[11]
